# Supplementary material for: Patterns of Intron Gain and Loss in Fungi
Source: PLoS Biol. 2004 Nov 30;2(12):e422. doi: 10.1371/journal.pbio.0020422 (PMC532390; doi:10.1371/journal.pbio.0020422)
Supplement: Table S1 — Also available at http://genes.mit.edu/NielsenEtAl/. (4.3 MB ZIP). [file pbio.0020422.st001.zip › NielsenEtAl/html/659.html]

AN6842.1.NCU01473.1.MG04007.1.FG09002.1


```
 CLUSTAL W (1.82) Multiple Sequence Alignments - Introns Inserted


Sequence 1: MG04007.1	499 aa
Sequence 2: FG09002.1	442 aa
Sequence 3: NCU01473.1	431 aa
Sequence 4: AN6842.1	418 aa
Alignment Length: 504 aa
Number Identitical Residues: 111 aa
Alignment Score (without introns) 7500


MG04007.1 	MSRCQQVGRTVTRCLRPQQHLAMATPSMSMASRSFSSTAGR~RDDVATTAIPPPENKNIS
NCU01473.1	MSKKKQVKRPLVRCLRQT-------------GPGAATCSTR2R-----------------
FG09002.1 	MSRCQVAMRPISRQLRPQ---------CPRIVRPFTSAAIR~R----------------T
AN6842.1  	MAHCERASRPIIQCLSKSSIRG--ISSVQLQTRAFQTTAAV~Q----------------E
          	*:: : . *.: : *   .  .   .:         : :   :                 

MG04007.1 	PARTPAAAKTTEASTGPAAAPSPPPASAAGQAAPAGSGSSDSQPAASTDGPTLAPKDPMV
NCU01473.1	VPPTTTTTSPVDSTTTPPPLPS----------------ATEAKATVADTVVG----GPEL
FG09002.1 	PAAETTAATATEADLDPNTVLP----------------EFEEQLMKSGKMP--------I
AN6842.1  	EAQTEAKSQPFYKAPDPALVSSP---------------RLERRLMRQGILP--------I
          	 .   : : .      *    ..                 : :                :

MG04007.1 	GSRRKQAAIAIADQSIPFAQLPFQCFQEARKVLHADRQEKIVQIVREAAKIKALEARDPE
NCU01473.1	GSRRRRAALATTG-NLPFEQLPYQCFQDARKILQQDRAAKIAQIVKETEKIKLIEARDAS
FG09002.1 	GSRRRRLAIRSTG-DLPFEHLPYQAFQEARKILSVDREEKLGKIQKEIEKISRLEAKKPE
AN6842.1  	GSRRRRAALQTTD-SLPFEQLPYQCFQEARKVLIADREEKLKEIATTSEKIAKIQALPEE
          	****:: *:  :. .:** :**:*.**:***:*  **  *: :*     **  ::*   .

MG04007.1 	TVTGGDRMKQMRLESMREYLNMLKVQADINDPEVKRRFEDGLG1DMNKPVYRHLAEQKWR
NCU01473.1	EFEGGEAAKQTRIKSLRKYIEELKILADINDPEVKRRFEDGRG1DMTKPVYRFMAERRWR
FG09002.1 	DIKGGQHIKDMRLRGLRKYVEELKILADANDPVVKKRFEDGTG1DMNKPIYRHYAEAKWR
AN6842.1  	EQT--KHSTYARLRALEVHLDRLKVLADINDPLVKKKFEDGQG1DMSKPIYRYLADQKWR
          	     .  .  *:..:. ::: **: ** *** **::**** * **.**:**. *: :**

MG04007.1 	GLPYRVIKQRIEQFSIIPDLLPKFEPVMDVQMSFRQRKTPPGTVVDSLTSEKPPTLRVQV
NCU01473.1	SMDYKIIAQRISQFHVVPDLLPAFDPTMDVKLSFRGYQVSPGAILDSRVTEVAPTLRMQV
FG09002.1 	SYDQRLINQRIHQFSIVPDVLPKLEPTADVQLYFRQLKVPPGQIVDSIVSEKAPRLRVQV
AN6842.1  	EYRRKILVQRITQMKVIPDVLPHCDPIVDTKLYFGRKAVPPGEFVNSQVSSVPPKLDIQV
          	    ::: *** *: ::**:**  :*  *.:: *    ..** .::* .:. .* * :**

MG04007.1 	FNKGERLVTVAVIDNDVPVVDKDGFARRCHFLAANIPIDPTTKAIPLMMIRDEQK-HLAV
NCU01473.1	FDKGERLLTVVVIDSDVPDVTHDNFKRRCHFLAANIPWDPSKTVLSLRSVGDRVEGDVGK
FG09002.1 	FDKGERLISIVVLDSDVPNPDSDTFNKRCHFLAANIPLSPNDTSLPLSRIKAED--QLAL
AN6842.1  	FDGENRLVTIAVVDSDVPNVEKDSFDYMMHYLAVNIPISALNTKVDLSQLSTES--QVVL
          	*:  :**:::.*:*.***    * *    *:**.*** ..  . : *  :  .   .:  

MG04007.1 	PWLPPFSQKGAPKHRLSIFIMEQKNNEIIDVAKLREAYIRSSNPDPAKNPNKFSIKSLKD
NCU01473.1	PWLPPFAQKGSPYHRLNVFVLEQKPGAKIDGEALKKHLE---------NRENFSLKGFRE
FG09002.1 	PWMPAFSQKGAPYHRLGIYLLEQEPGQKIDVAKLKKLYS---------NRDGFSLKSFRD
AN6842.1  	PWLPPVAQKGSPYHRLSLFVMEQKDNKPLDFAAVKAKET---------DRLNKLLRTLQA
          	**:*..:***:* ***.::::**: .  :*   ::             :     :: :: 

MG04007.1 	KFPALEPVGFNIFRTEWDEYTADVMARNNIPGADVEFKHARVHSMKPERGPRGWEAKRQG
NCU01473.1	KFD-LEPVGFNLFRSEWDEGTAEVMERHGIPGAEVEFKRQKFASLKPPRKARGWEAKRQK
FG09002.1 	KFN-TTPFGFNMFRSVWDENTAAVMARSNIPGSDIEFRPTRVHSLKPPVKPRGWEAKRQG
AN6842.1  	RYH-LKPIGAHLFRTIWDDDTRDVMKEIGFPLADMELRRKRSEALPYKR--------RNP
          	::    *.* ::**: **: *  ** . .:* :::*::  :  ::            *: 

MG04007.1 	PKYRHLWKYTHRIA--TPKRKFVK--
NCU01473.1	PKYKSLWKYVKRIA------------
FG09002.1 	PKYRHLWKYTKNIKGISNSRGWIKRR
AN6842.1  	STFR----------------------
          	..::
```
